# Supplementary figures and images for: HOXC6 promotes migration, invasion and proliferation of esophageal squamous cell carcinoma cells via modulating expression of genes involved in malignant phenotypes
Source: PeerJ. 2019 Mar 14;7:e6607. doi: 10.7717/peerj.6607 (PMC6421064; doi:10.7717/peerj.6607)

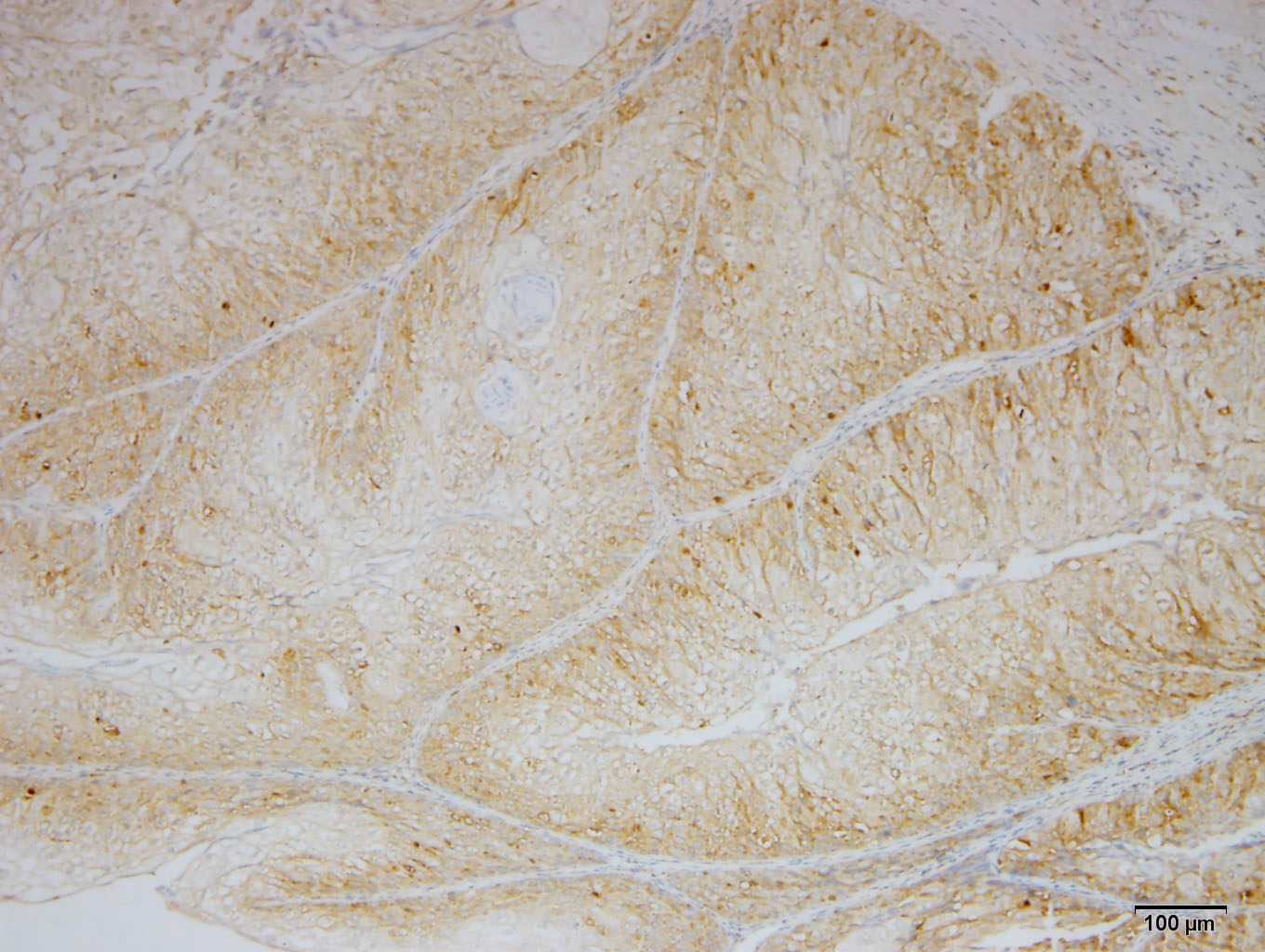

Supplement: Supplemental Information 1 [file peerj-07-6607-s001.zip › raw data/Figure 2/Fig 2A-100(+).jpg]

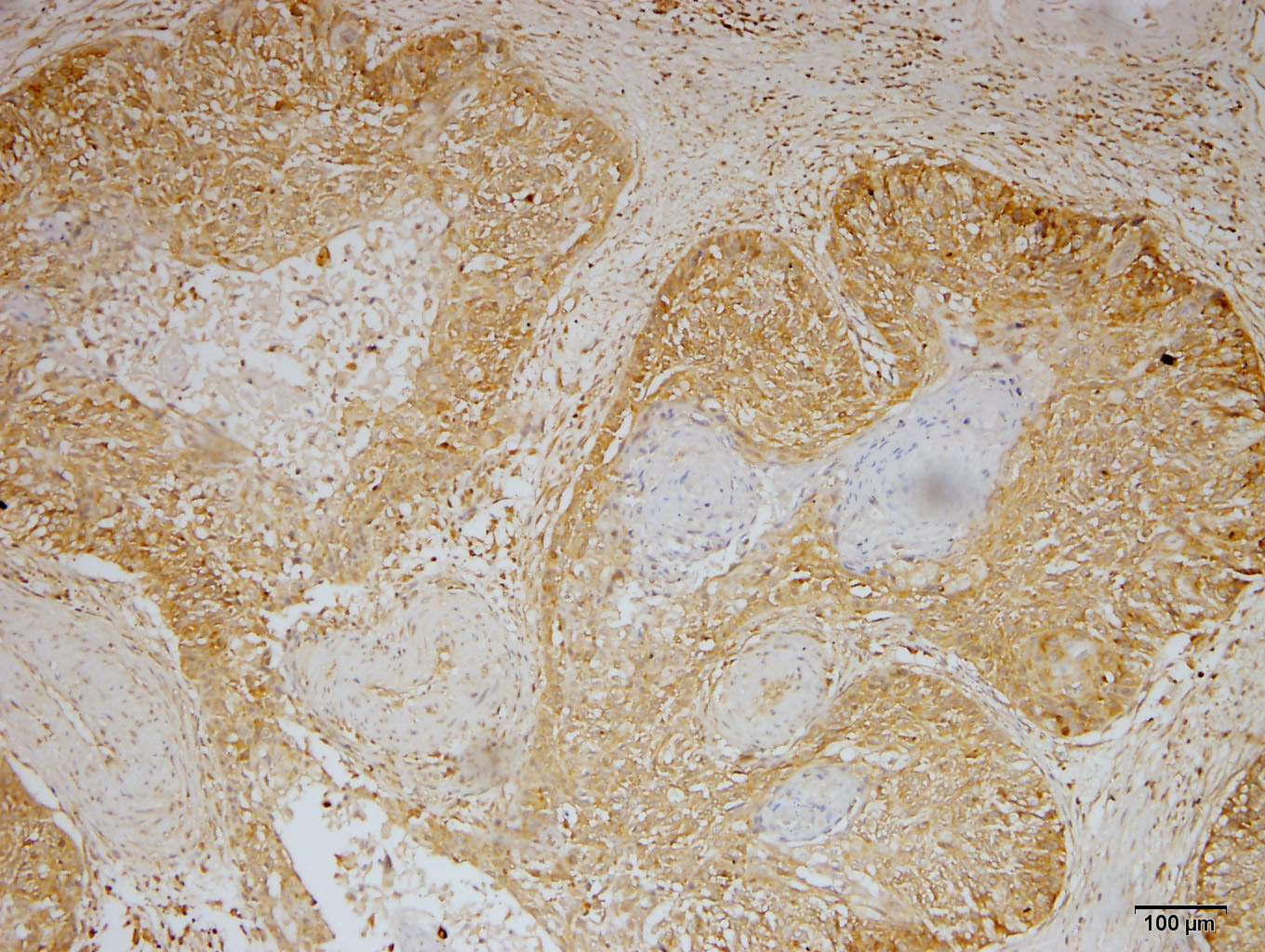

Supplement: Supplemental Information 1 [file peerj-07-6607-s001.zip › raw data/Figure 2/Fig 2A-100(++).jpg]

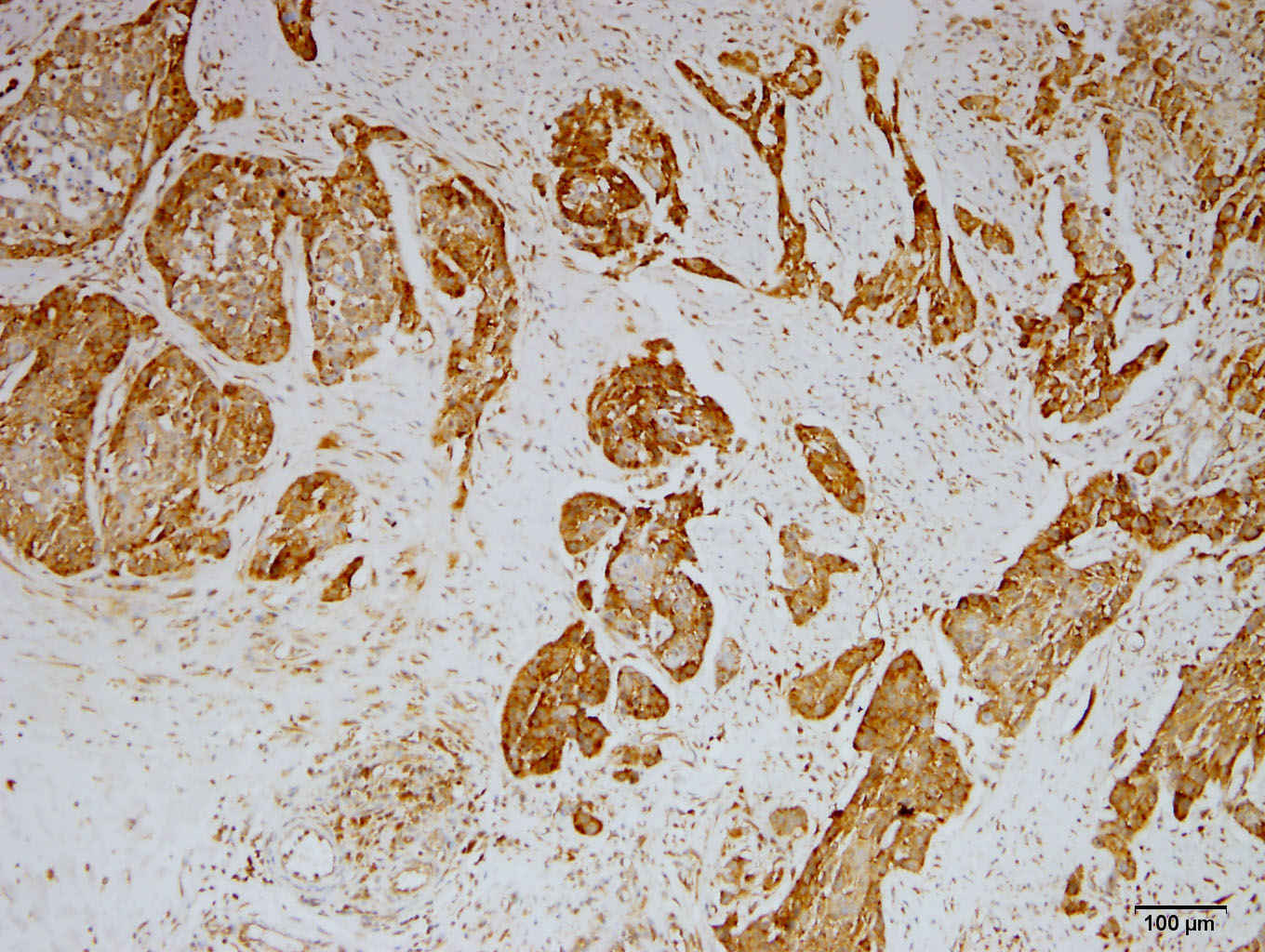

Supplement: Supplemental Information 1 [file peerj-07-6607-s001.zip › raw data/Figure 2/Fig 2A-100(+++).jpg]

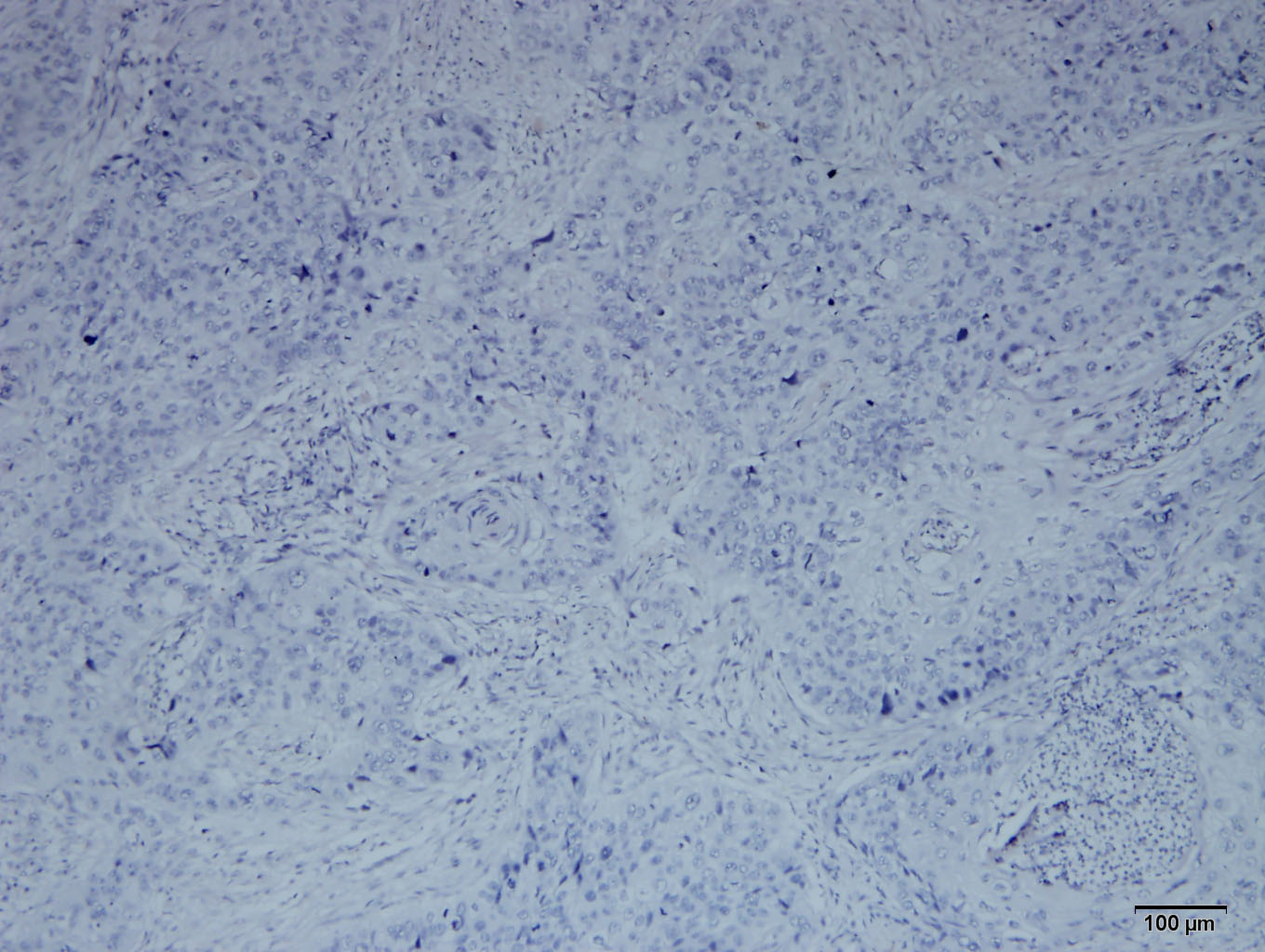

Supplement: Supplemental Information 1 [file peerj-07-6607-s001.zip › raw data/Figure 2/Fig 2A-100(-).jpg]

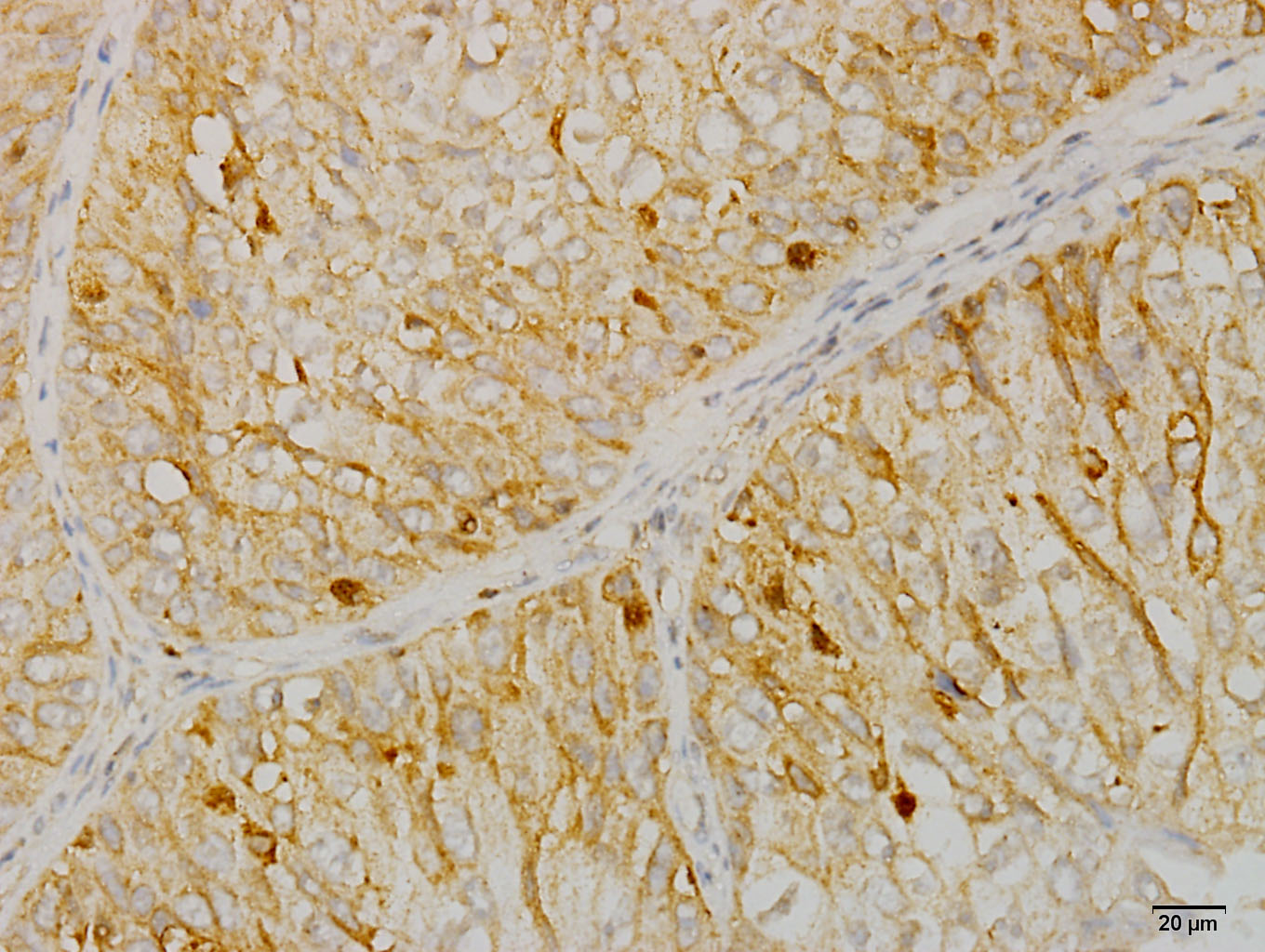

Supplement: Supplemental Information 1 [file peerj-07-6607-s001.zip › raw data/Figure 2/Fig 2A-400(+).jpg]

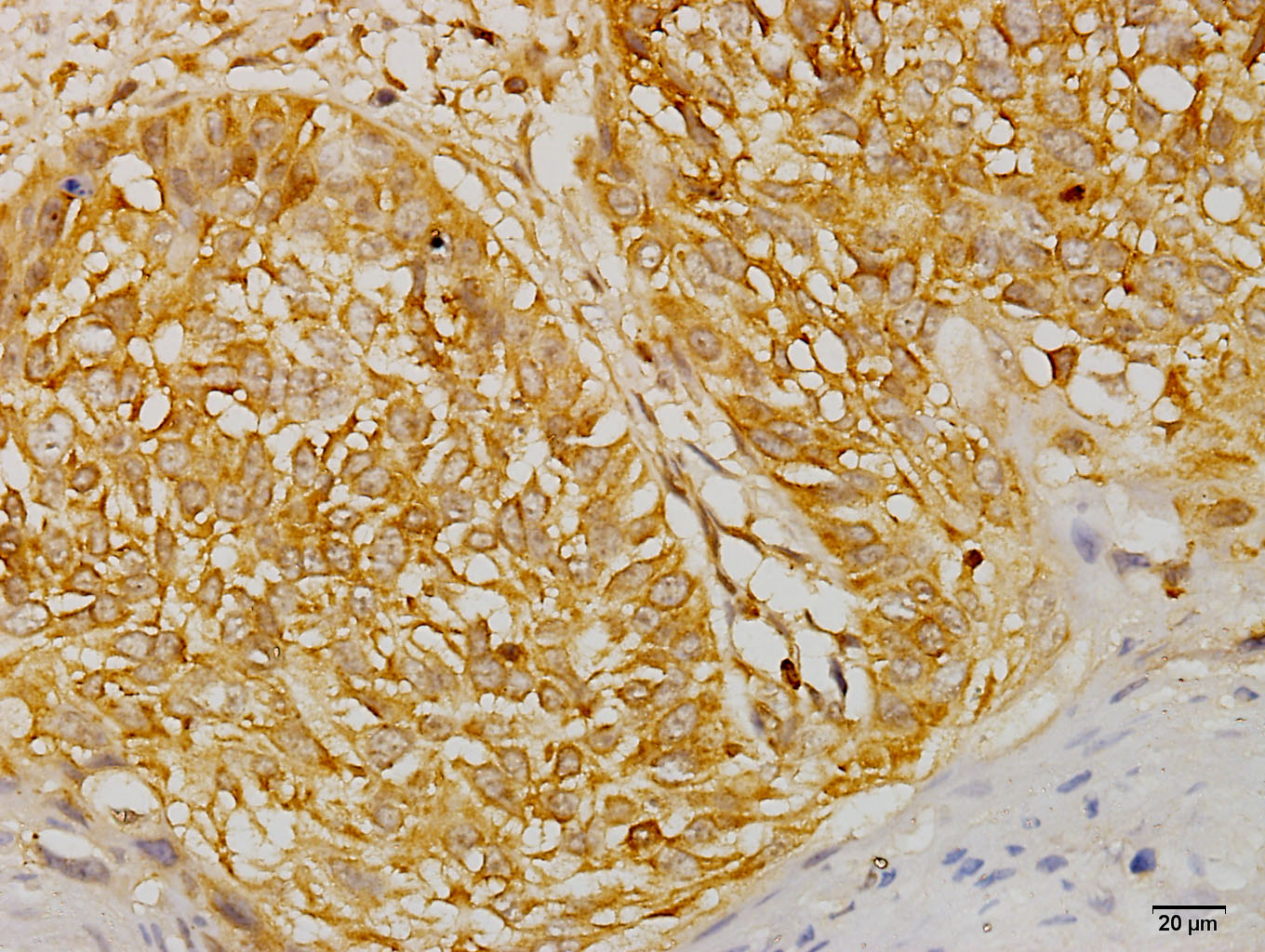

Supplement: Supplemental Information 1 [file peerj-07-6607-s001.zip › raw data/Figure 2/Fig 2A-400(++).jpg]

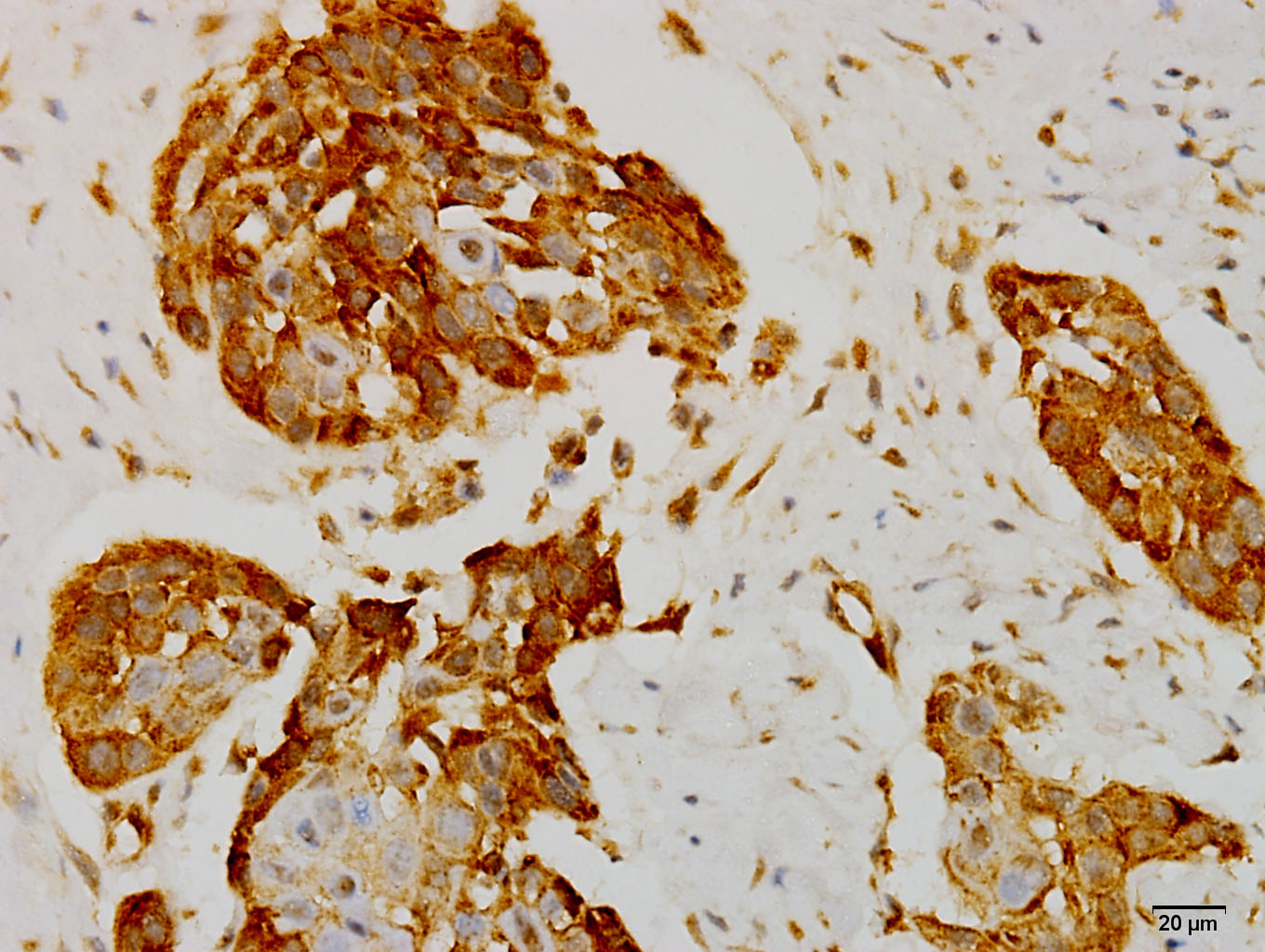

Supplement: Supplemental Information 1 [file peerj-07-6607-s001.zip › raw data/Figure 2/Fig 2A-400(+++).jpg]

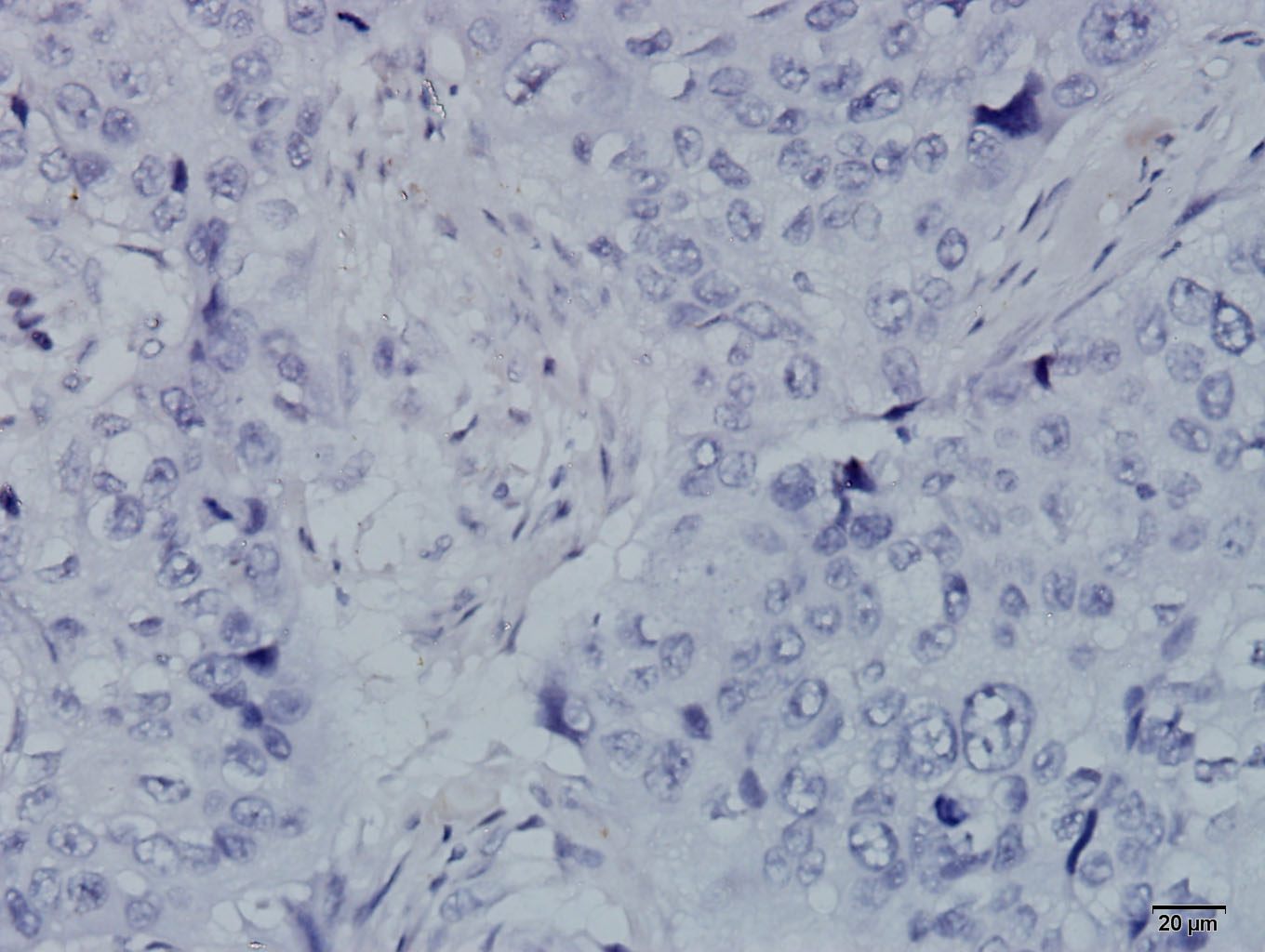

Supplement: Supplemental Information 1 [file peerj-07-6607-s001.zip › raw data/Figure 2/Fig 2A-400(-).jpg]

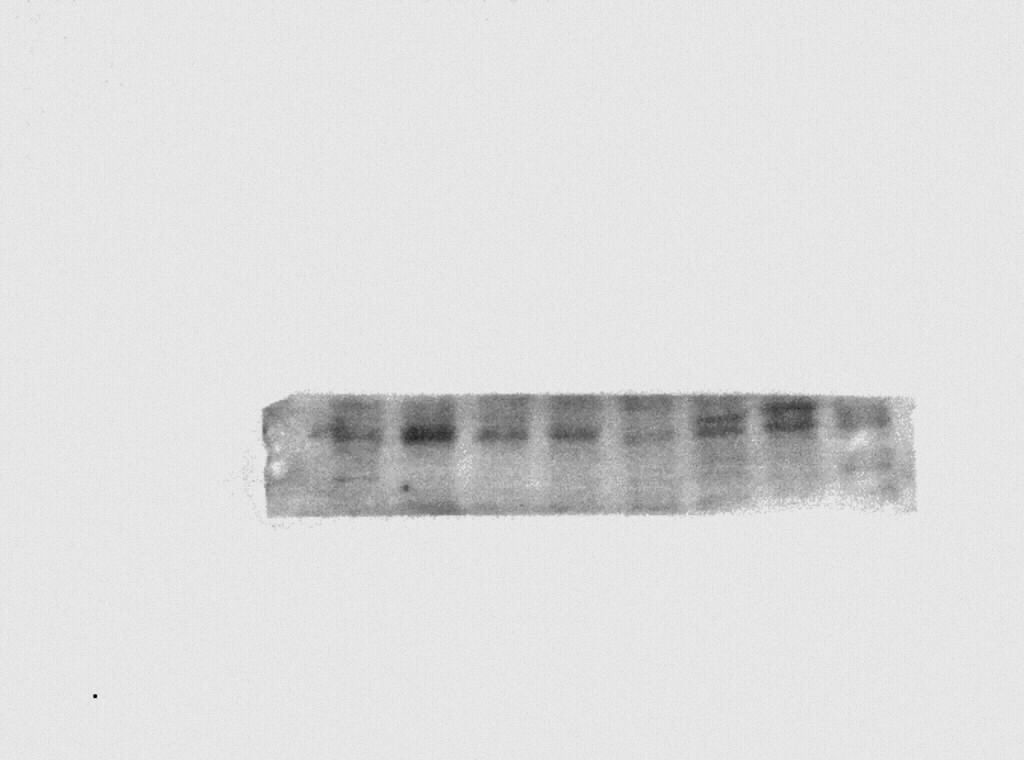

Supplement: Supplemental Information 1 [file peerj-07-6607-s001.zip › raw data/Figure 2/Figure 2D-HOXC6(from lane1 to 6 belongs to tissues of ESCC patients, lane 7 and 8 belongs to other samples).tif]

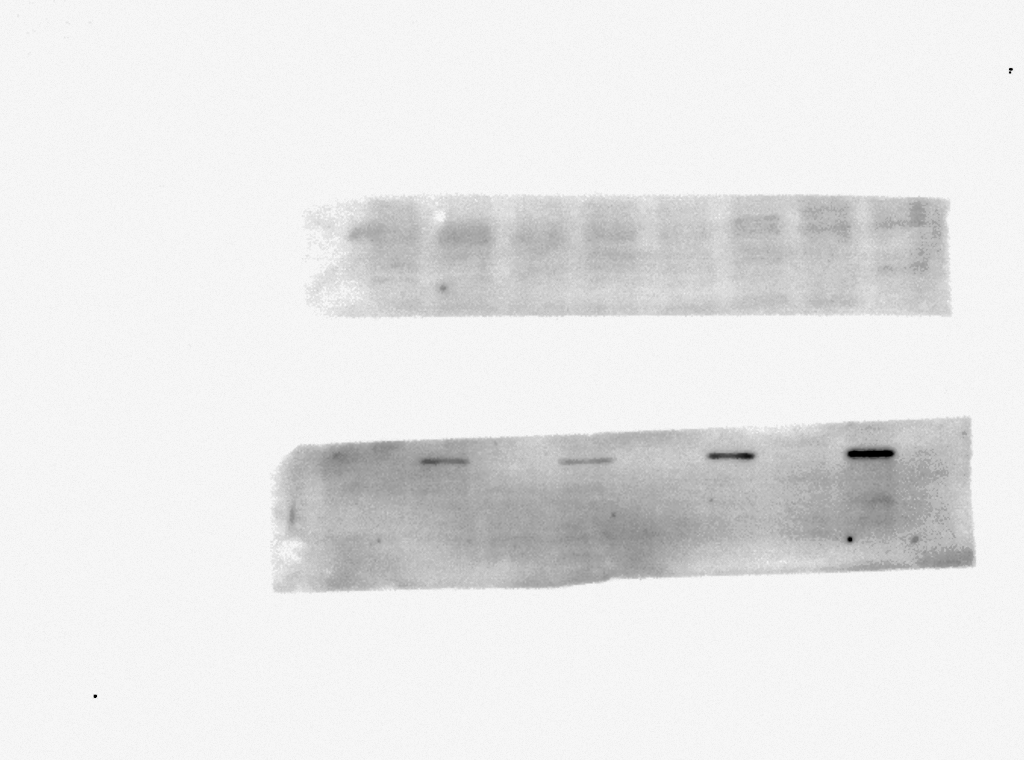

Supplement: Supplemental Information 1 [file peerj-07-6607-s001.zip › raw data/Figure 3/Fig 3C-HOXC6( below image, lane1 to lane 4 belongs to stable cells, lane 5 to lane 9 belongs to other samples).tif]

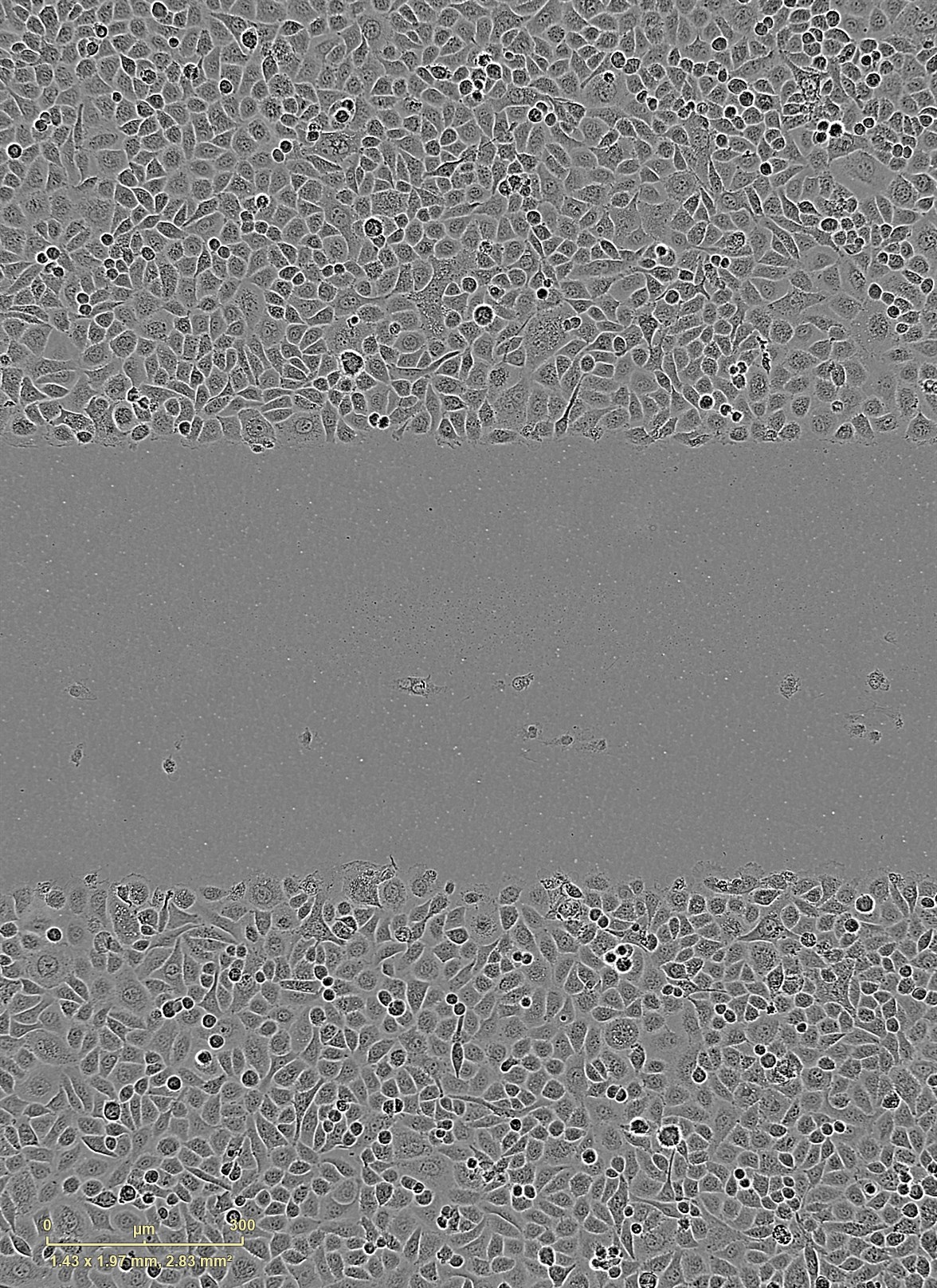

Supplement: Supplemental Information 1 [file peerj-07-6607-s001.zip › raw data/Figure 4/Fig 4A-Eca109-HOXC6-0h.jpg]

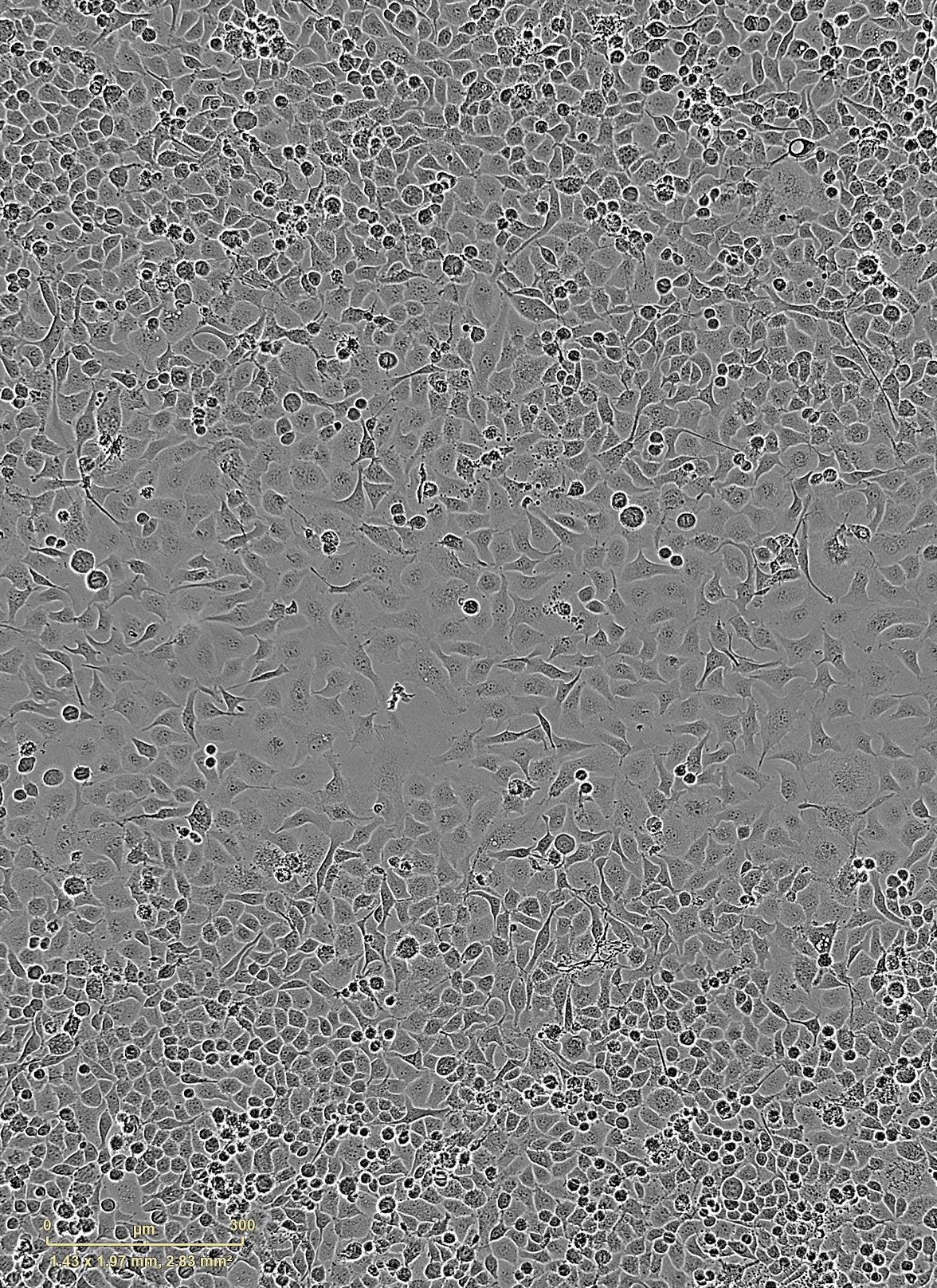

Supplement: Supplemental Information 1 [file peerj-07-6607-s001.zip › raw data/Figure 4/Fig 4A-Eca109-HOXC6-18h.jpg]

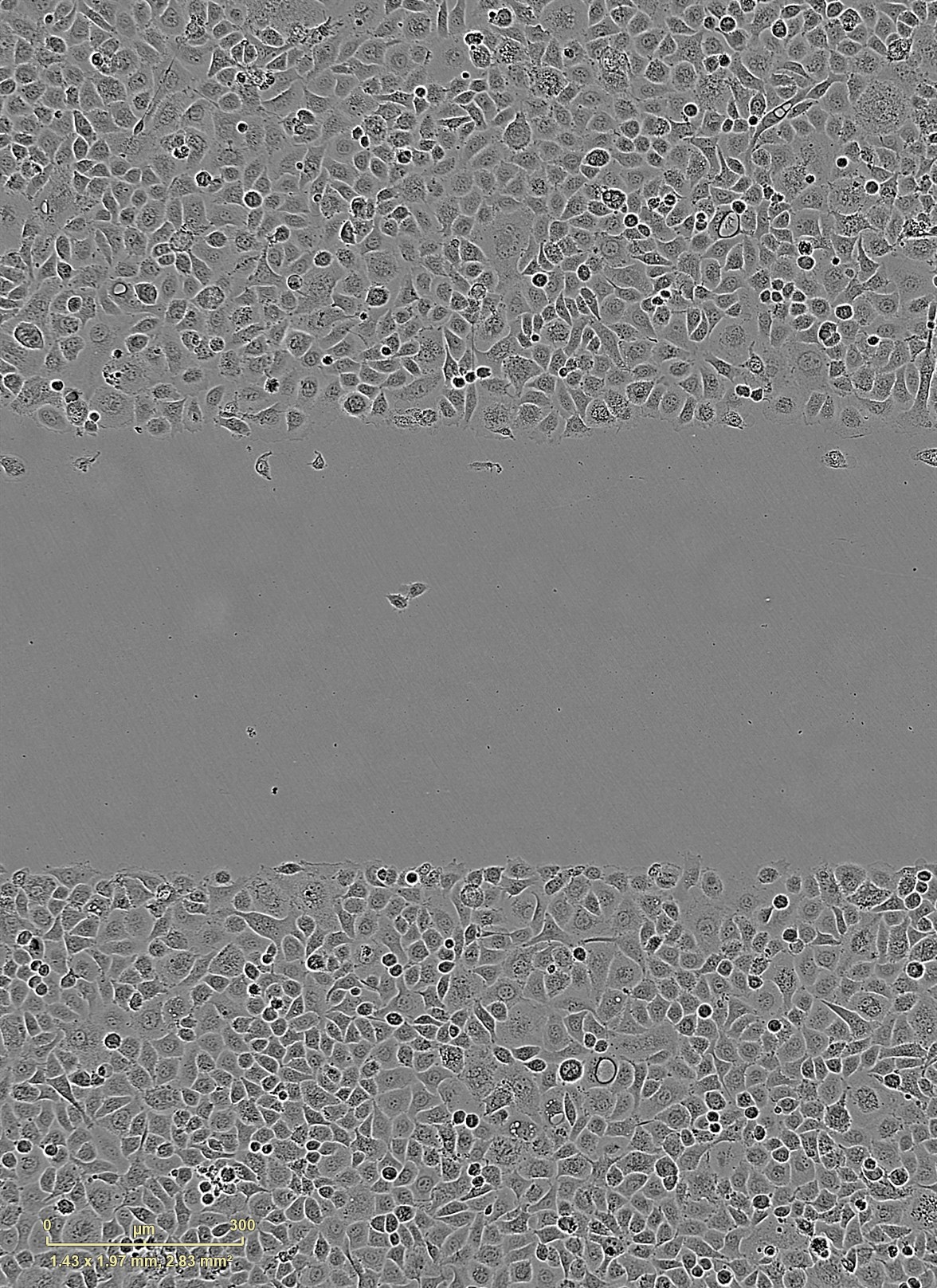

Supplement: Supplemental Information 1 [file peerj-07-6607-s001.zip › raw data/Figure 4/Fig 4A-Eca109-NEO-0h.jpg]

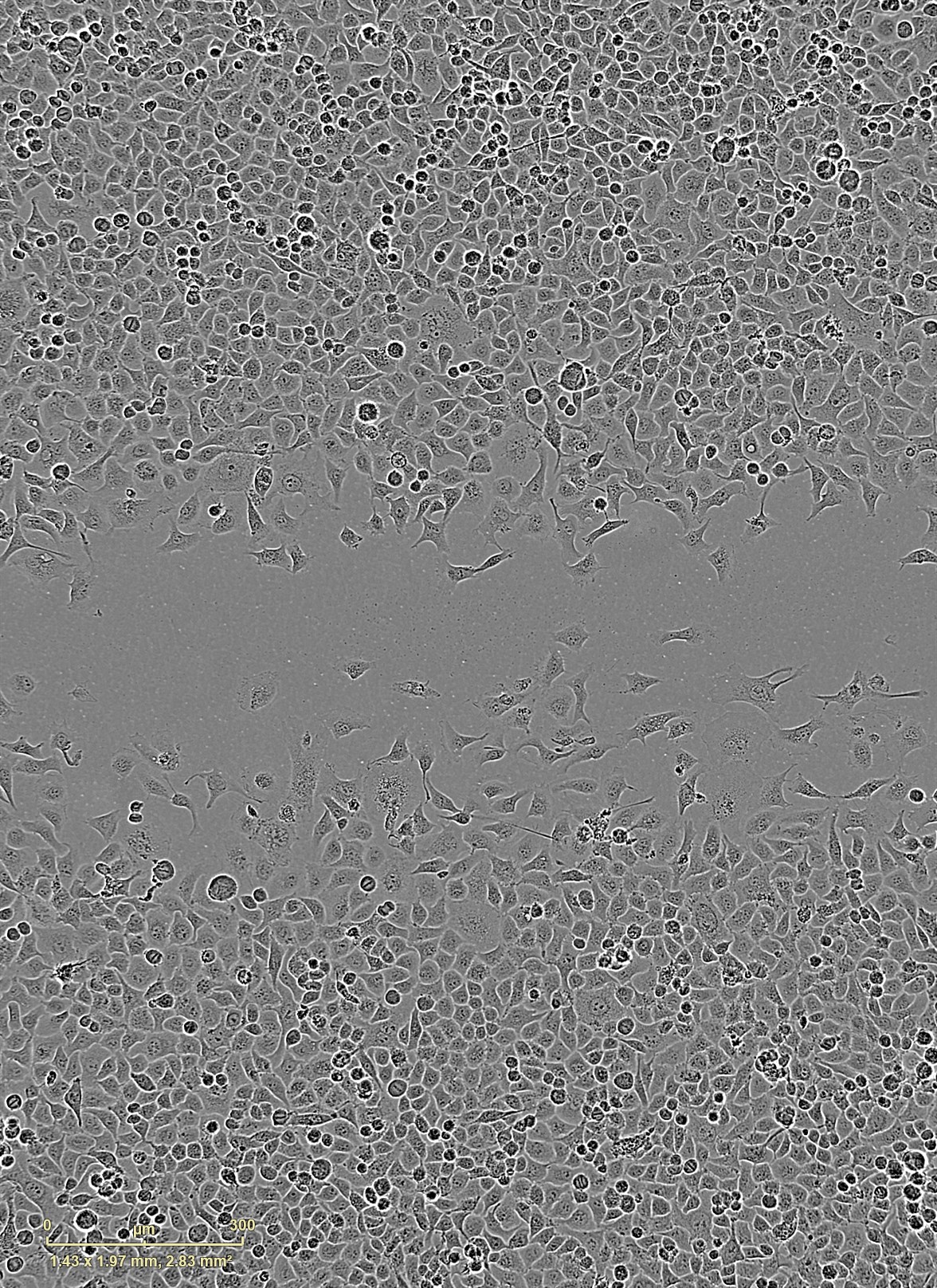

Supplement: Supplemental Information 1 [file peerj-07-6607-s001.zip › raw data/Figure 4/Fig 4A-Eca109-NEO-18h.jpg]

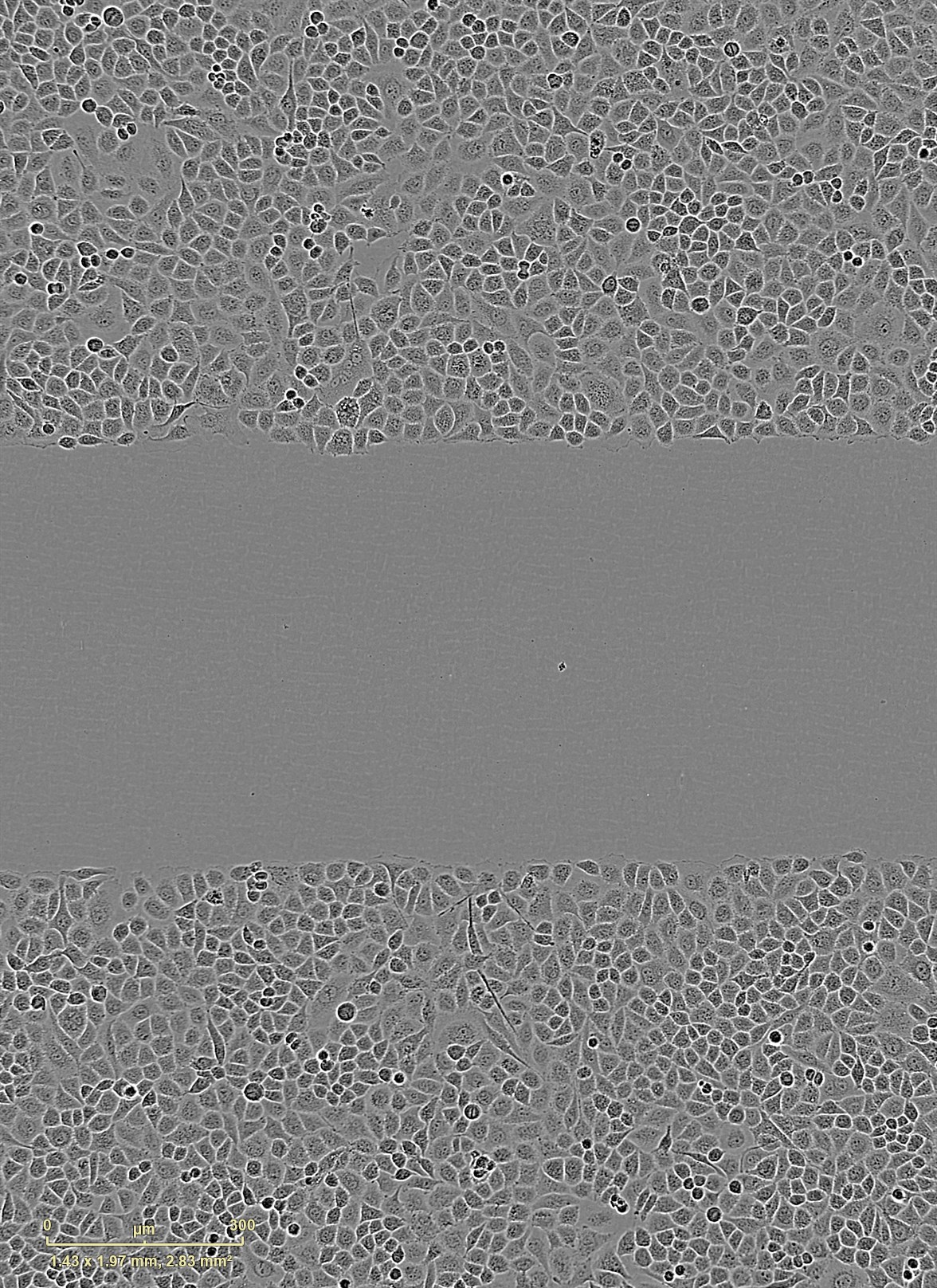

Supplement: Supplemental Information 1 [file peerj-07-6607-s001.zip › raw data/Figure 4/Fig 4B-TE10-HOXC6-0h.jpg]

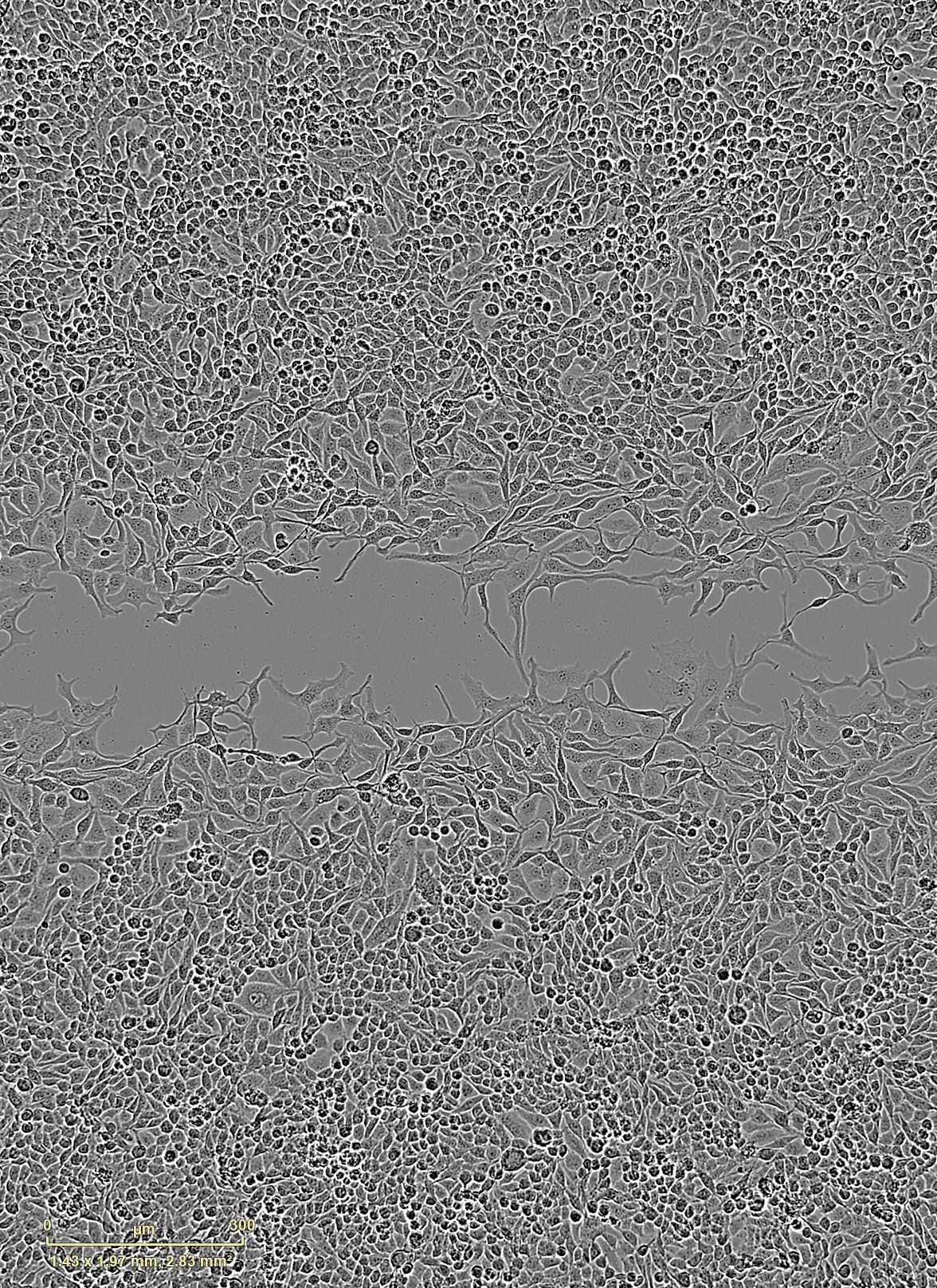

Supplement: Supplemental Information 1 [file peerj-07-6607-s001.zip › raw data/Figure 4/Fig 4B-TE10-HOXC6-28h.jpg]

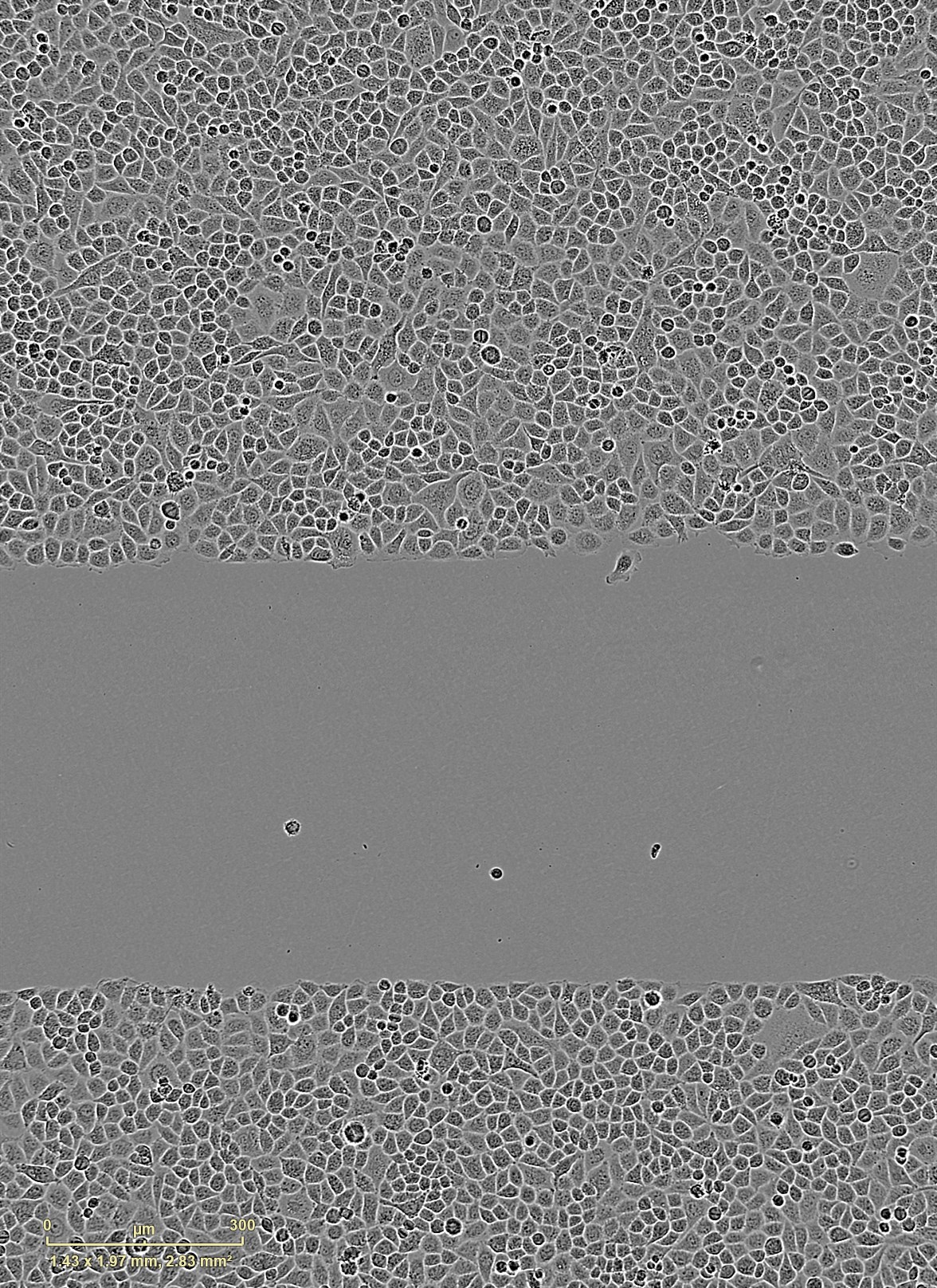

Supplement: Supplemental Information 1 [file peerj-07-6607-s001.zip › raw data/Figure 4/Fig 4B-TE10-NEO-0h.jpg]

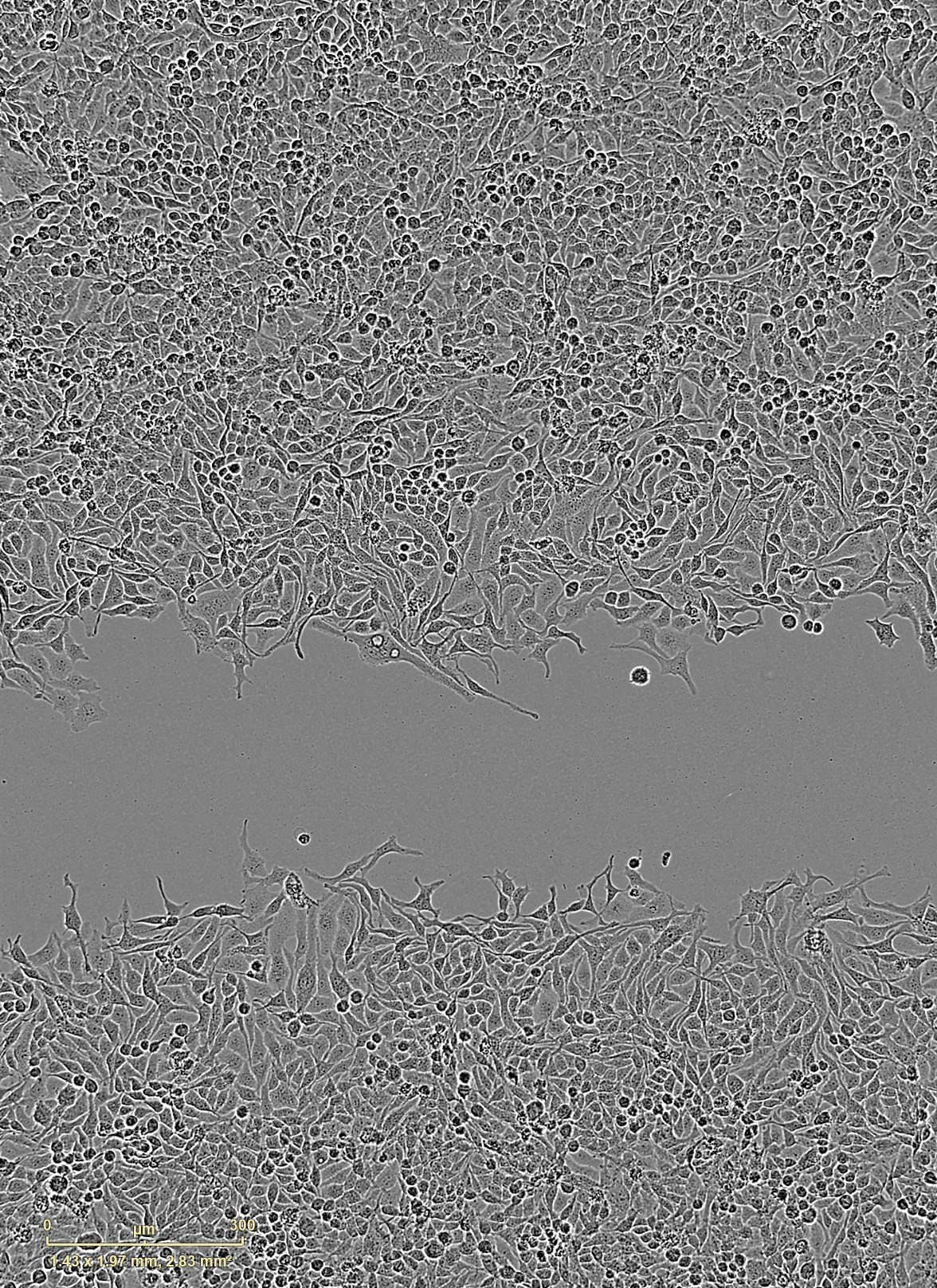

Supplement: Supplemental Information 1 [file peerj-07-6607-s001.zip › raw data/Figure 4/Fig 4B-TE10-NEO-28h.jpg]

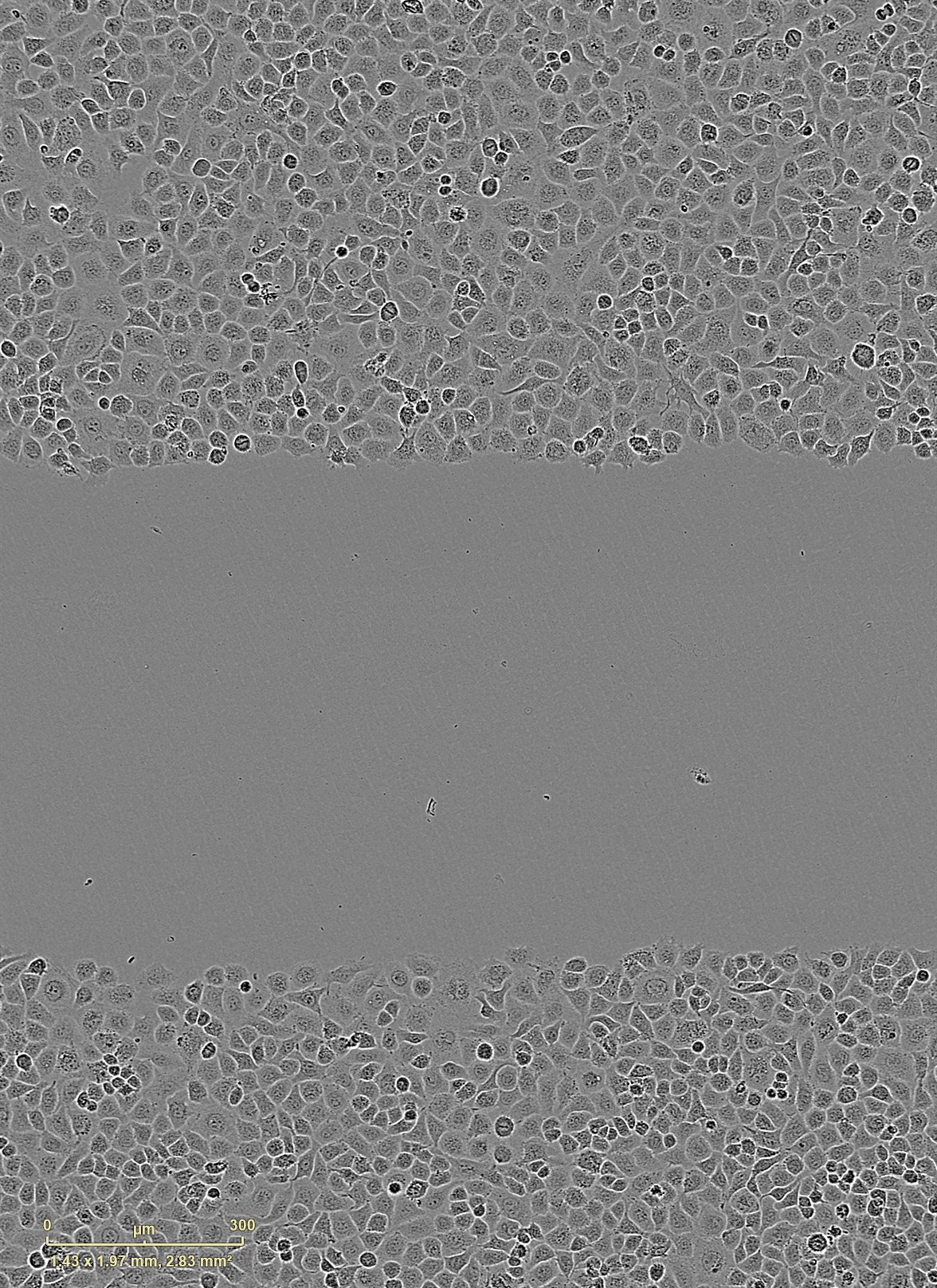

Supplement: Supplemental Information 1 [file peerj-07-6607-s001.zip › raw data/Figure 5/Fig 5A-Eca109-HOXC6-0h.jpg]

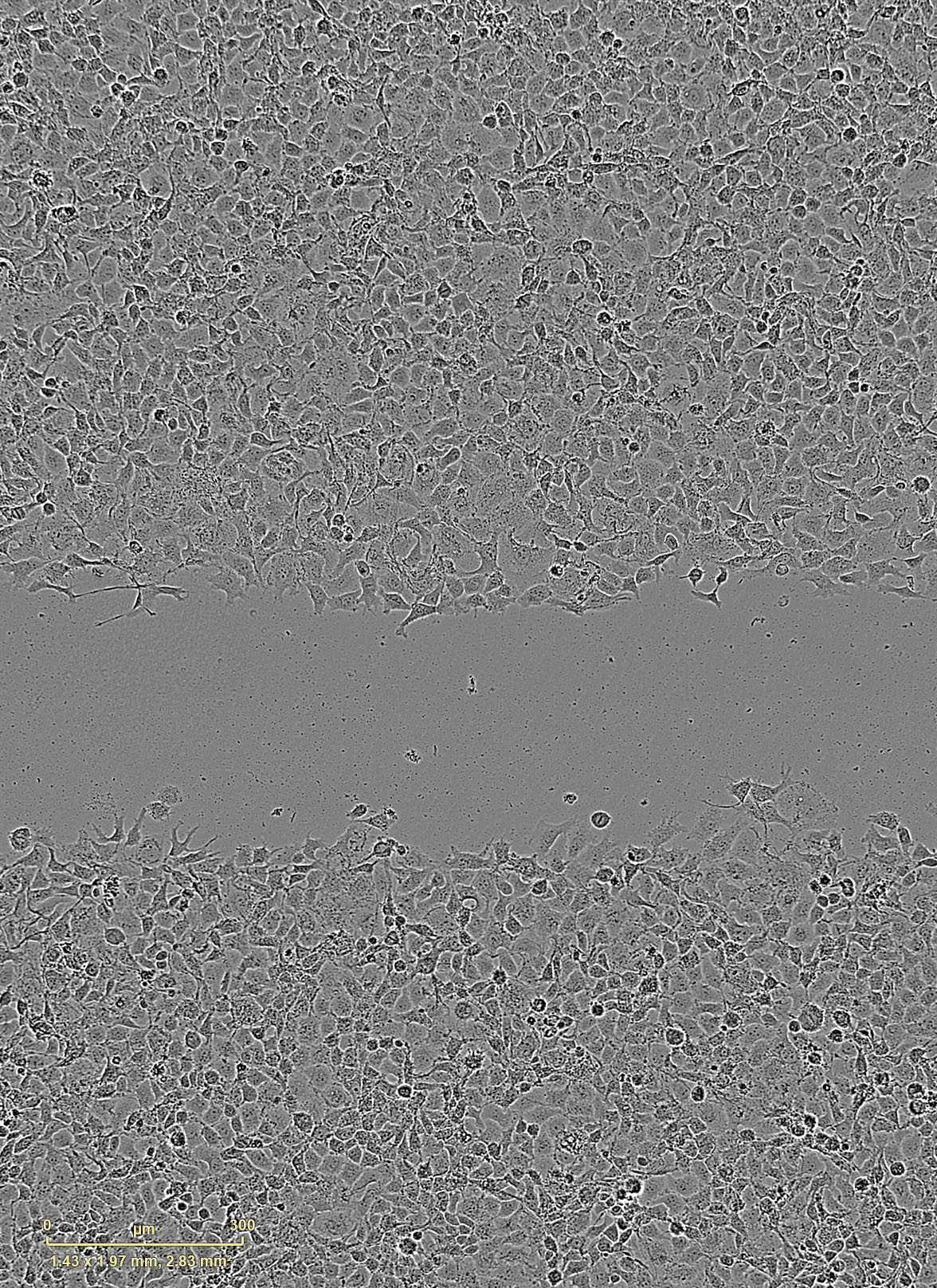

Supplement: Supplemental Information 1 [file peerj-07-6607-s001.zip › raw data/Figure 5/Fig 5A-Eca109-HOXC6-24h.jpg]

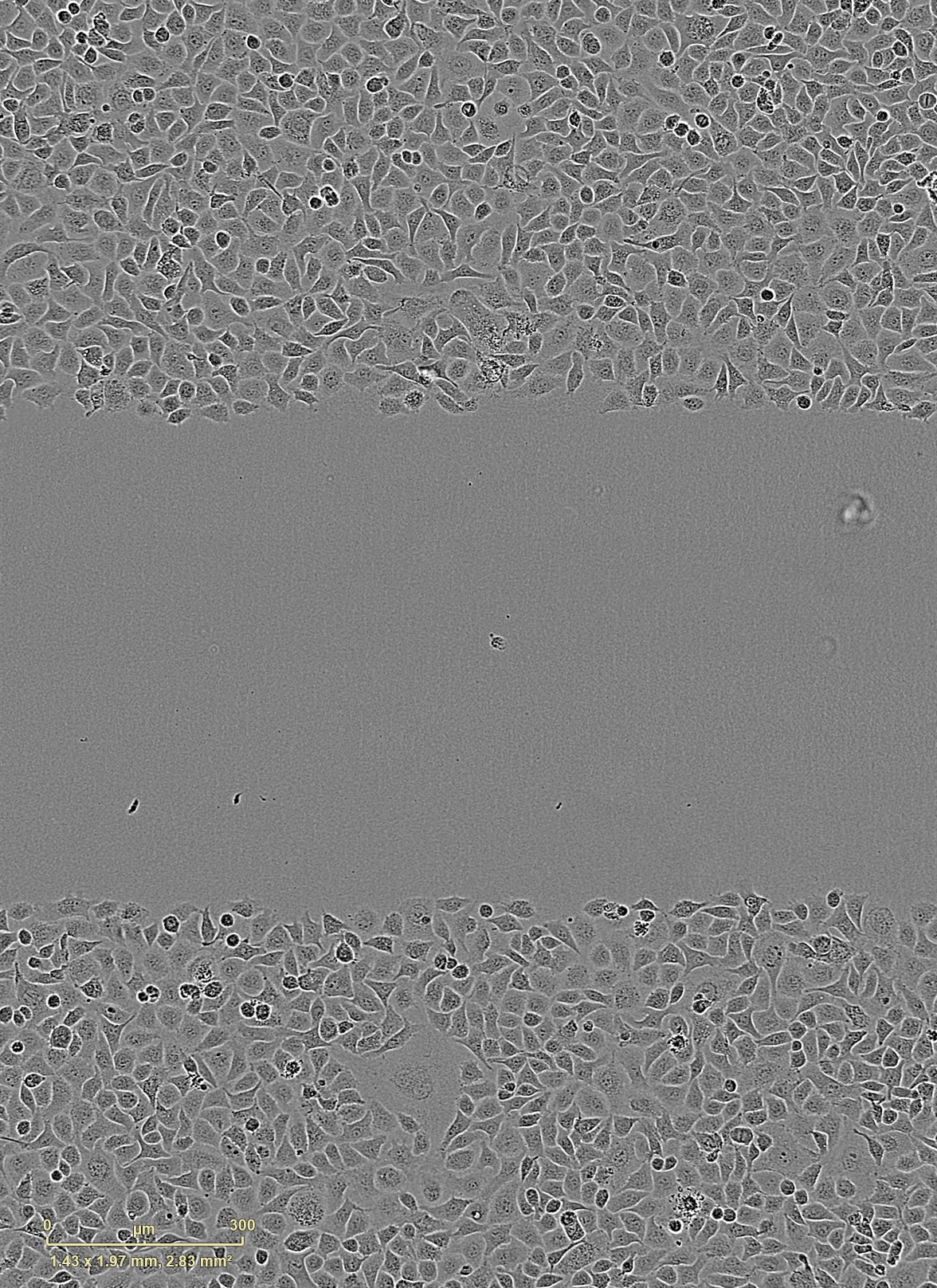

Supplement: Supplemental Information 1 [file peerj-07-6607-s001.zip › raw data/Figure 5/Fig 5A-Eca109-NEO-0h.jpg]

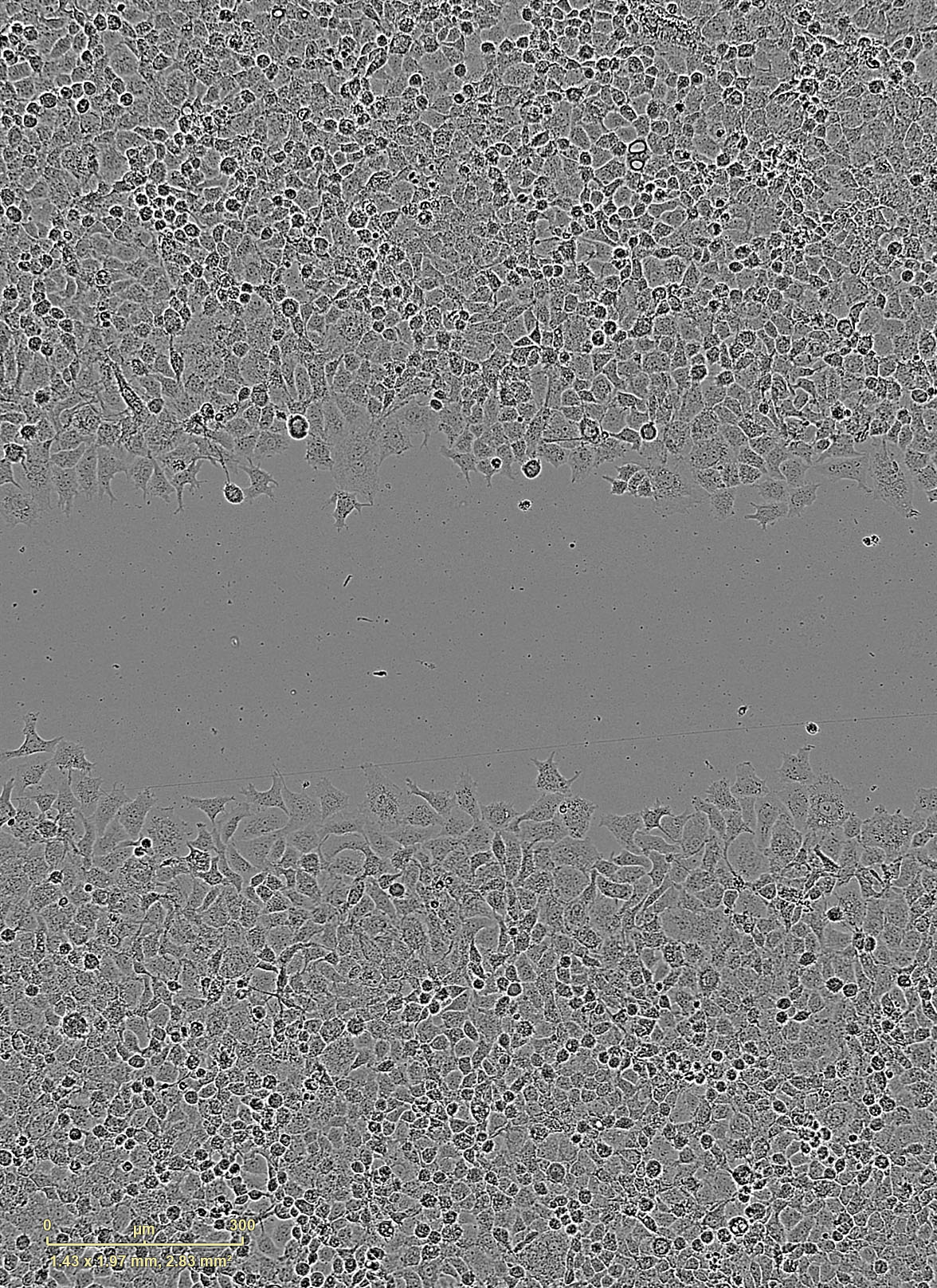

Supplement: Supplemental Information 1 [file peerj-07-6607-s001.zip › raw data/Figure 5/Fig 5A-Eca109-NEO-24h.jpg]

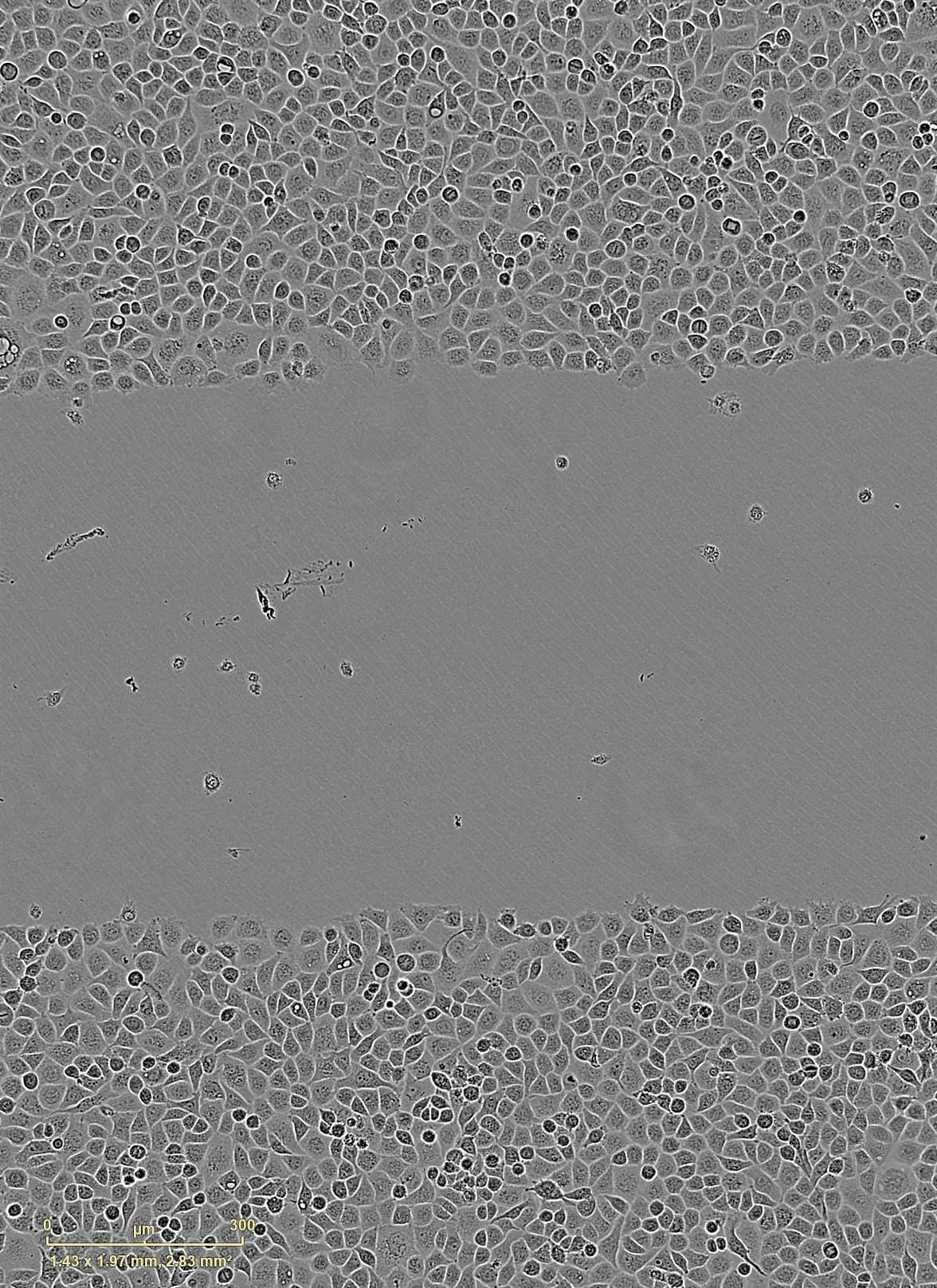

Supplement: Supplemental Information 1 [file peerj-07-6607-s001.zip › raw data/Figure 5/Fig 5B-TE10-HOXC6-0h.jpg]

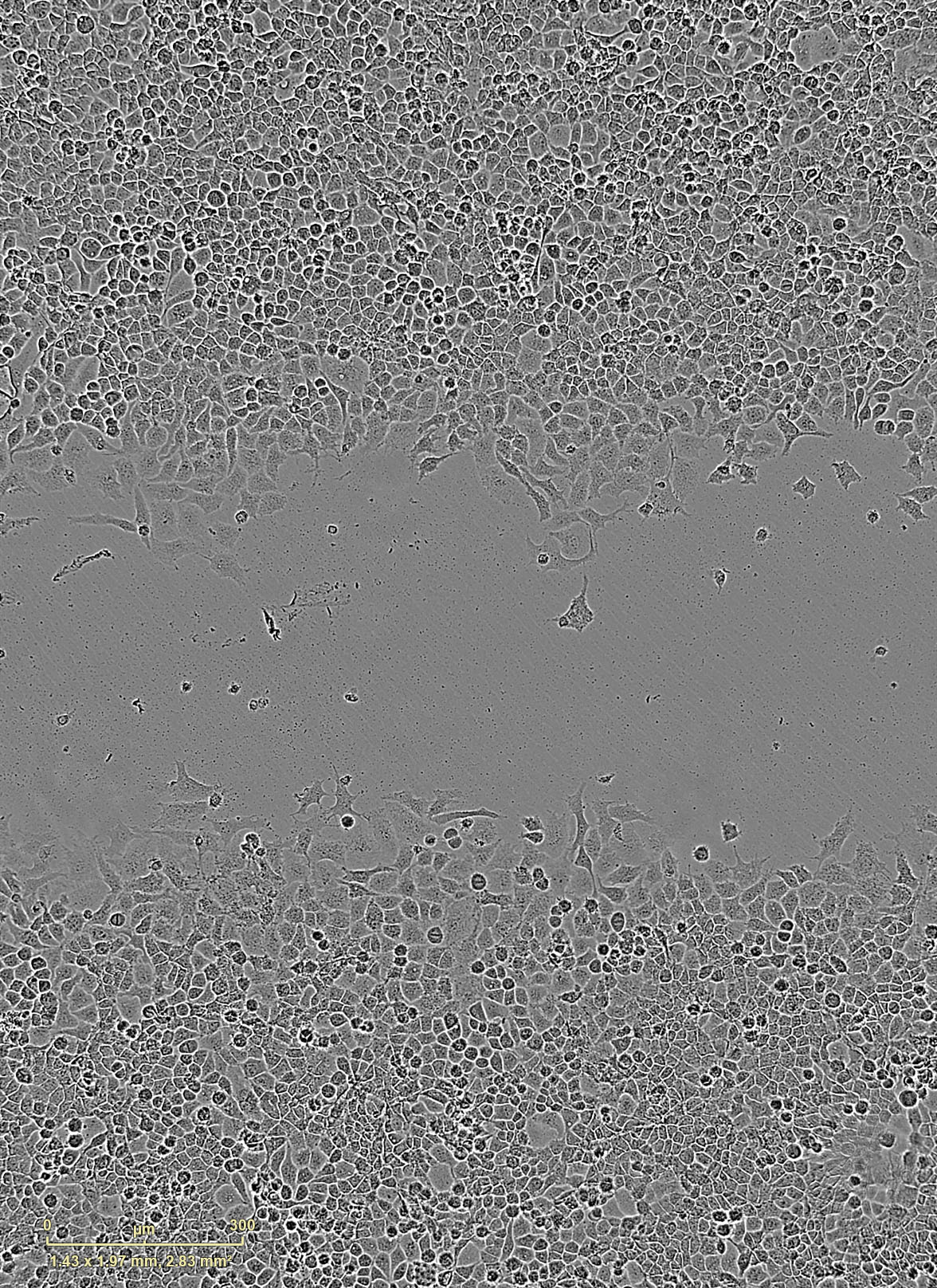

Supplement: Supplemental Information 1 [file peerj-07-6607-s001.zip › raw data/Figure 5/Fig 5B-TE10-HOXC6-22h.jpg]

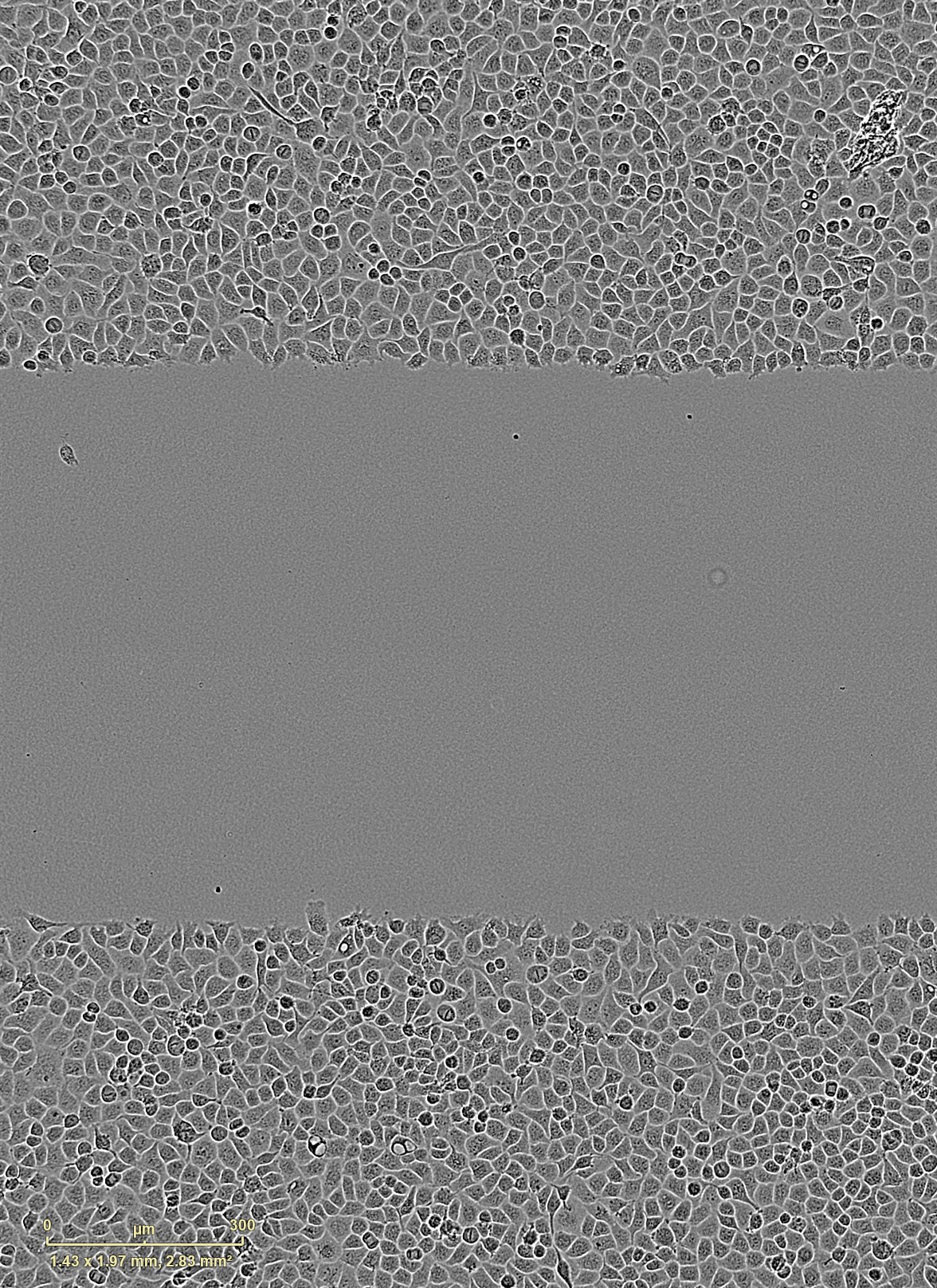

Supplement: Supplemental Information 1 [file peerj-07-6607-s001.zip › raw data/Figure 5/Fig 5B-TE10-NEO-0h.jpg]

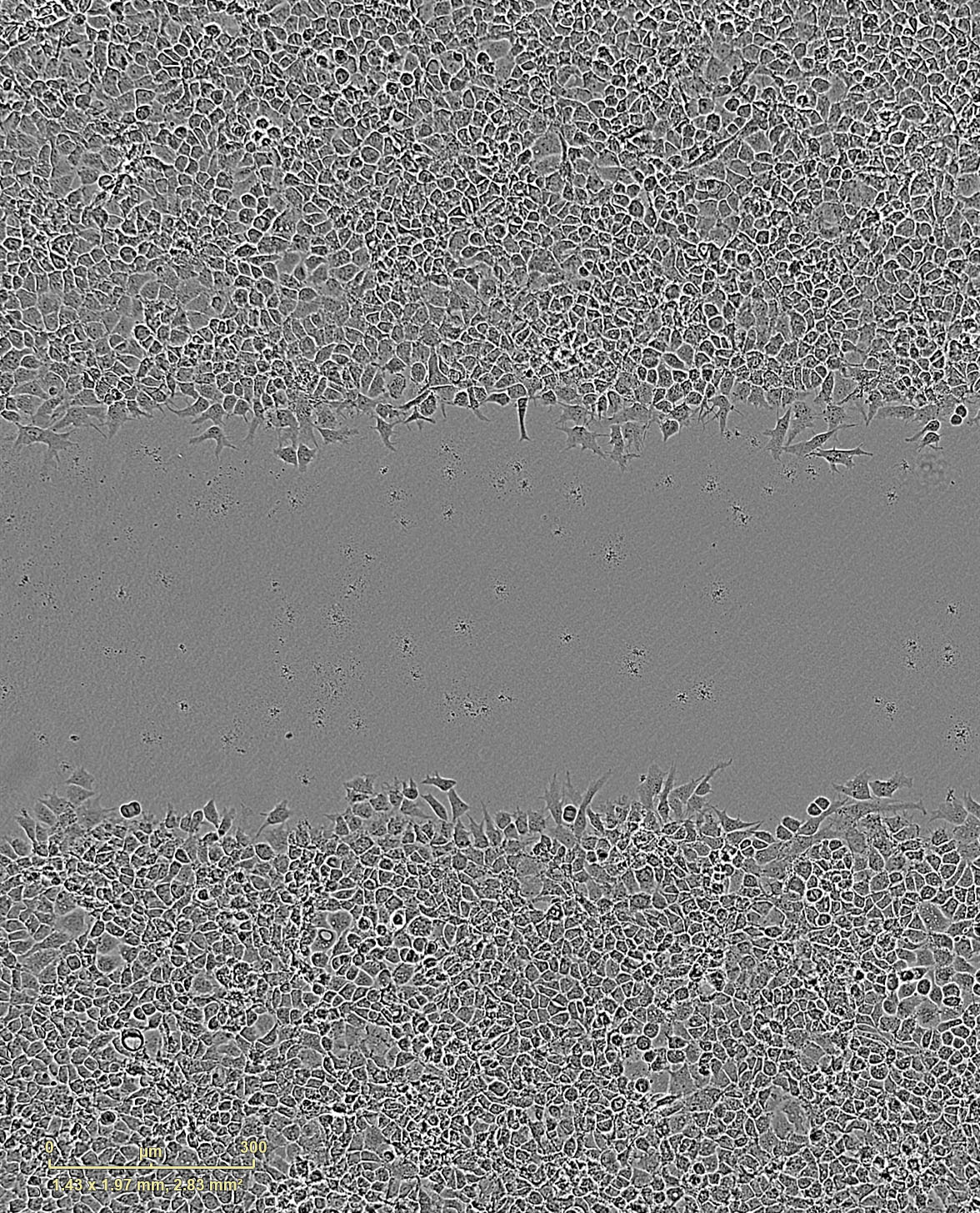

Supplement: Supplemental Information 1 [file peerj-07-6607-s001.zip › raw data/Figure 5/Fig 5B-TE10-NEO-22h.jpg]

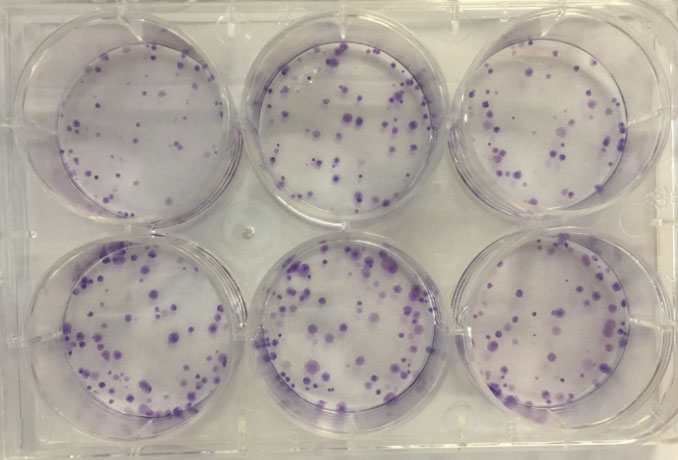

Supplement: Supplemental Information 1 [file peerj-07-6607-s001.zip › raw data/Figure 6/Fig 6C-Eca109.jpg]

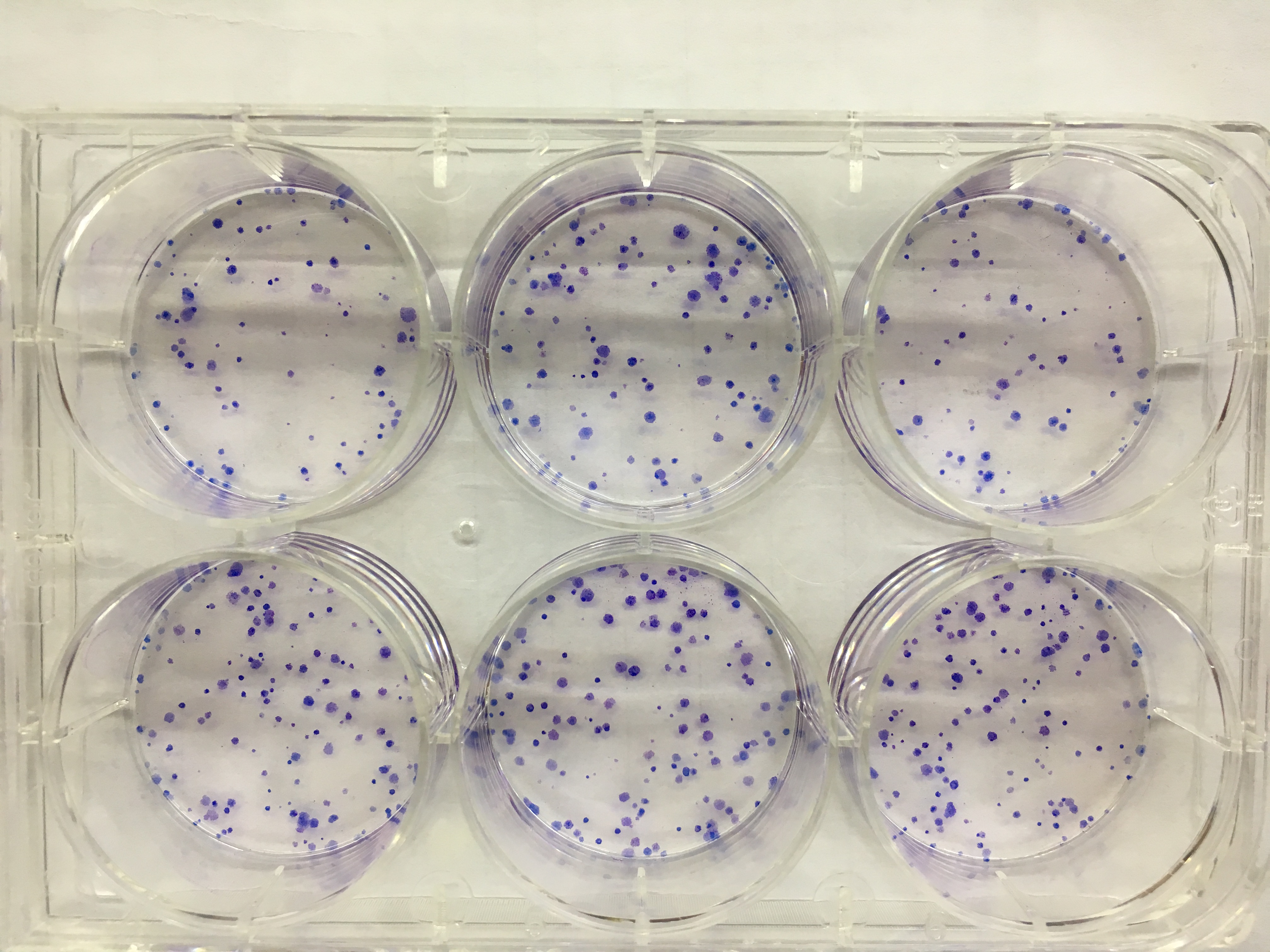

Supplement: Supplemental Information 1 [file peerj-07-6607-s001.zip › raw data/Figure 6/Fig 6C-TE10.JPG]
